# International Rare Cancers Initiative (IRCI)

**DOI:** 10.3332/ecancer.2013.ed20

**Published:** 2013-05-21

**Authors:** Nicola Keat, Kate Law, Andrea McConnell, Matthew Seymour, Jack Welch, Ted Trimble, Denis Lacombe, Anastassia Negrouk

**Affiliations:** 1 Cancer Research UK, Angel Building, 407 St John Street, London, EC1V 4AD, UK; 2 NCRN, University of Leeds, Leeds, UK; 3 Cancer Therapy Evaluation Program, Division of Cancer Treatment and Diagnosis, NCI, Bethesda, MD, USA; 4 Center for Global Health, NCI, Bethesda, MD, USA; 5 European Organisation for Research and Treatment of Cancer, Brussels, Belgium (DL, AN)

We declare that we have no conflicts of interest.

There is no internationally agreed definition of a rare cancer. In Europe, rare diseases are often defined as those with a prevalence of <50/100,000 [1]. In the US, the Orphan Drug Designation Program defines rare diseases as those affecting <200,000 people in the total US population, equivalent to a prevalence of 64/100,000 [1]. The problem with both these definitions is that they are based on prevalence, which does not properly reflect the health burden of incident diseases such as cancer. RARECARE more usefully defines rare cancers as those with an incidence of <6/100,000/year [3]. Using this definition, the combined annual incidence rate of all rare cancers in Europe is about 108 per 100,000, corresponding to 541,000 new diagnoses annually or 22% of all cancer diagnoses. This is more than any single common cancer. And returning to prevalence, about 4,300,000 patients are living today in the European Union with a diagnosis of a rare cancer, 24% of the total cancer prevalence.

Unfortunately, the average outcome for patients with a rare cancer is inferior to those with more common cancers [1]. One factor contributing to this is the lack of evidence upon which to base treatment, so in an attempt to address this issue, the International Rare Cancers Initiative (IRCI) was established early in 2011. IRCI is a joint initiative between the National Institute for Health Research Cancer Research Network (NCRN), Cancer Research UK (CR-UK), the National Cancer Institute (NCI) and the European Organisation for Research and Treatment of Cancer (EORTC).

The objective of this initiative is to facilitate the development of international clinical trials for patients with rare cancers in order to boost the progress of new treatments for these patients. For the purpose of IRCI, ‘rare’ has been broadly defined as an incidence of <2 per 100,000. Occasionally, rare clinical scenarios with less rare cancers have also been considered, e.g. relapsed anal cancer. To date, IRCI has excluded rare molecular sub-types of common cancers; however, a rare molecular sub-type could be considered if it is a distinct, prospectively identifiable rare sub-group with a strong rationale for separate research, rather than inclusion as a molecular stratum in a mainline trial. Priority has been given to cancers with potential for an interventional trial (usually randomised) rather than an audit, registry or non-trial tissue collection. The initiative hopes to encourage the use of innovative methodologies, such as Multi-Arm Multi-Stage (MAMS) trial designs and Bayesian statistics, to maximise the potential for answering research questions and to identify and overcome barriers to international trials to allow agreed IRCI trials to run smoothly.

At the outset of the initiative clinical communities associated with each partner organisation were asked to identify rare cancers where there was enthusiasm for international collaborations and the potential for development of an interventional clinical trial. It was not possible to take forward every rare cancer type suggested, but where interests coincided in at least two of the partner organisations, and the IRCI Board could see potential for research development, IRCI groups were formed. The following nine rare cancers have formed the core activities of IRCI to date: Salivary gland cancer, Anaplastic thyroid cancer, Small bowel adenocarcinoma, Gynaecological sarcoma, Fibrolamellar hepatocellular carcinoma, Penile cancer, Thymoma, Ocular melanoma, Relapsed/metastatic anal cancer.

IRCI organises face-to-face meetings and teleconferences to allow potential clinical trial designs to be discussed and developed. Wherever possible these face-to-face meetings are run alongside international conferences that the leads and other experts are already likely to be attending. IRCI has been met with considerable enthusiasm by the international clinical community.

Of the initial nine groups taken on by IRCI, seven are actively developing 10 clinical trials for submission to appropriate funding bodies. The IRCI Gynaecological Sarcoma Group has made great progress and in September 2012 the first IRCI study opened to recruitment - A phase III randomised trial of gemcitabine plus docetaxel followed by doxorubicin versus observation for uterus-limited, high grade uterine leiomyosarcoma (IRCI 001, ClinicalTrials.gov registration number NCT01533207). This IRCI study is led by Dr Martee Hensley (Memorial Sloan-Kettering Cancer Centre, MSKCC) and co-ordinated by the Gynecologic Oncology Group (GOG). It opened to recruitment at the MSKCC early in September 2012 and it is hoped that the first patient will be recruited to this study imminently. The study is currently being processed by the EORTC and Glasgow Clinical Trials Unit and is due to open to recruitment in Europe in the next few months.

Ultimately, IRCI aims to open and recruit to international clinical trials for patients with rare cancers in order to boost the progress of new treatments for these patients and ultimately, improve outcomes. IRCI aims to do this by continuing to aid its existing groups, working through the issues of trial setup with the trials developed and taking on further rare cancer types (it is expected that two new rare cancer groups will be initiated in 2013 - relapsed Ewing’s sarcoma and Desmoplastic small round cell tumours). Additionally, IRCI may expand to involve other interested organisations. Finally, developing relationships with industry is a key objective.
